# Female Sex but Not Oestrogen Receptor Expression Predicts Survival in Advanced Gastroesophageal Adenocarcinoma—A Post-hoc Analysis of the GO2 Trial

**DOI:** 10.3390/cancers15092591

**Published:** 2023-05-03

**Authors:** Mark A. Baxter, Lindsay C. Spender, Shaun Walsh, Susan Bray, Gemma Skinner, Sharon King, Peter S. Hall, Matthew J. Seymour, Russell D. Petty, on behalf of the GO2 Investigators

**Affiliations:** 1 Division of Molecular and Clinical Medicine, Ninewells Hospital and Medical School, University of Dundee, Dundee DD1 4HR, UK; 2 Tayside Cancer Centre, Ninewells Hospital and Medical School, NHS Tayside, Dundee DD2 1SY, UK; 3 Department of Pathology, Ninewells Hospital and Medical School, NHS Tayside, Dundee DD2 1SY, UK; 4 Cancer Research UK Edinburgh Centre, MRC Institute of Genetics & Molecular Medicine, The University of Edinburgh, Edinburgh EH4 2XR, UK; 5 Leeds Institute of Medical Research at St James’, University of Leeds, Woodhouse, Leeds LS2 9JT, UK; 6 Leeds Teaching Hospitals NHS Trust, Beckett Street, Leeds LS9 7TF, UK

**Keywords:** gastroesophageal cancer, oestrogen receptor, older adults, prognosis, biomarker

## Abstract

**Simple Summary:**

Gastroesophageal adenocarcinoma (GOA) is a cancer that has poor survival. Most cases are diagnosed when a cure is not possible, and treatment often has many side effects. It occurs less often and is associated with better outcomes in females. The reason for this is not known. We sought to use samples from a clinical trial in older adults with GOA to investigate whether this observation could be related to oestrogen and its action through oestrogen receptors. We found no clear link between outcome and oestrogen receptor expression but did note improved survival with older age and female sex.

**Abstract:**

Gastroesophageal adenocarcinoma is a disease of older adults that is associated with a very poor prognosis. It is less common and has better outcomes in females. The reason for this is unknown but may relate to signalling via the main oestrogen receptors (ER) α and β. In this study, we sought to investigate this using the GO2 clinical trial patient cohort. GO2 recruited older and/or frail patients with advanced gastroesophageal cancer. Immunohistochemistry was performed on tumour samples from 194 patients. The median age of the population was 76 years (range 52–90), and 25.3% were female. Only one (0.5%) tumour sample was positive for ERα, compared to 70.6% for ERβ expression. There was no survival impact according to ERβ expression level. Female sex and younger age were associated with lower ERβ expression. Female sex was also associated with improved overall survival. To our knowledge, this is the largest study worldwide of ER expression in a cohort of patients with advanced gastroesophageal adenocarcinoma. It is also unique, given the age of the population. We have demonstrated that female sex is associated with better survival outcomes with palliative chemotherapy but that this does not appear to be related to ER IHC expression. The differing ER expression according to age supports the concept of a different disease biology with age.

## 1. Introduction

Gastroesophageal adenocarcinoma (GOA) is increasing in incidence in the Western world, and the prognosis remains poor, with a 5-year survival of approximately 20% [1,2]. Despite recent advances in systemic therapy, prognosis in the advanced setting remains less than a year in biomarker-negative patients [3]. This figure is lower in older and/or frail patients [4]. There is an urgent need to identify biomarkers to accurately assess disease biology and prognostic outcomes.

It is well documented epidemiologically that in GOA, there is a male predilection, which narrows post-menopausally (~11:1 at age 50–54, ~4:1 at age 75–79) [5,6]. The reason for this has not been established; however, it does not appear to be related to female reproductive [7] or traditional risk factors [8]. It has been proposed that the endocrine milieu that occurs in pre- and peri-menopausal females may be protective.

The epidemiological observation of a protective role for oestrogen in the development of gastroesophageal cancer is supported by studies in both oesophageal and gastric cancer investigating the use of hormone replacement therapy (HRT) and tamoxifen [9,10,11], as well as the protective effect of breastfeeding [12]. This effect is not specific to females. In men, higher levels of circulating dehydroepiandrosterone, oestradiol and free oestradiol also appear protective for the development of both oesophageal and gastric adenocarcinoma [13]. Together, these observations have led to the suggestion that oestrogen may confer an anti-tumour effect, which warrants further investigation. This is supported by the observation of improved survival for females with gastroesophageal adenocarcinoma [14,15,16].

The exact mechanisms underlying this effect remain unclear; however, they may be mediated through oestrogen receptor (ER) signalling. The main ERs are ERα (ESR1) and ERβ (ESR2) [17], which are differentially expressed according to the organ; ERα is predominantly expressed in female sex organs, while ERβ is widely expressed in other tissues, including the oesophagus and stomach [18].

To date, most studies investigating the immunohistochemical expression of ERα and ERβ in GOA have focused on tumour samples from younger patients in the curative setting. There is, therefore, limited data in the advanced setting or in an older population. This is important as GOA is a disease of older age, and there is increasing evidence of differing biomarker expression and disease biology with age in other tumour groups [19]. ERs can be targeted with existing therapies, and immunohistochemistry (IHC) is easy to perform; therefore, their prognostic role also warrants investigation in this setting.

To address this knowledge gap, we utilised stored tumour samples from the GO2 trial. The GO2 trial recruited older and/or frailer patients with advanced gastroesophageal cancer, felt to better represent real-world patients encountered in clinical practice [4]. The trial sought to investigate the role of chemotherapy dose de-escalation in this population. In this post hoc study, we investigated tumour immunohistochemical expression and the prognostic role of ERα and ERβ in an older, advanced GOA population treated with palliative chemotherapy.

## 2. Materials and Methods

### 2.1. Study Cohort

The GO2 trial recruited 559 patients, including both squamous and non-squamous histology [4]. Patients were randomised to either a ‘likely to benefit’ (*n =* 514) or ‘uncertain to benefit’ (*n =* 45) arm. In the ‘likely to benefit’ arm, patients were randomised to either 100% (Level A), 80% (Level B) or 60% (Level C) doublet chemotherapy regimen of oxaliplatin/capecitabine. 100% dose was oxaliplatin 130 mg/m^2^ on day 1 and capecitabine 625 mg/m^2^ twice daily on days 1–21, on a 21-day cycle. In the ‘uncertain to benefit’ arm, patients were randomised to either Level C or supportive care alone. This study focuses on the adenocarcinoma population only.

The GO2 trial clinical database is held at the Clinical Trials Research Unit at Leeds Institute of Clinical Trials Research, University of Leeds, Leeds. All data analysis in this manuscript uses this anonymised dataset.

Between November 2014 and January 2018, formalin-fixed paraffin-embedded (FFPE) tumour blocks were collected from 395/559 (70.7%) patients in the GO2 trial. Biospecimen collection was an optional part of the GO2 trial design from the outset and included in the ethical trial approval (REC Number 13/YH/0229). These samples were initially collected and resided within the NHS Grampian Biorepository (REC Number 16/NS/0055). No more samples were collected as part of this study. The samples were also registered with the NHS Tayside Biorepository (REC approval 17/ES/0130). Following the transfer of tissue, all analysis was performed in NHS Tayside.

### 2.2. Immunohistochemistry

All sectioning and IHC were performed by the NHS Tayside Biorepository, and slides were provided for scoring. Antigen retrieval and de-paraffinisation were performed using DAKO EnVision™ FLEX Target Retrieval solution (high pH) buffer in a DAKO PT Link. Sections were blocked in Flex Peroxidase-Blocking Reagent and incubated overnight at 4 °C with anti-Estrogen Receptor Beta 14C8 (ab288 Abcam, Cambridge, UK) at a dilution of 1 in 500 and anti-Estrogen Receptor Alpha (SP1 Ventana, Tucson, AZ, USA) at a dilution of 1 in 50. Immunostaining using the DAKO EnVision™ FLEX system (Agilent Technologies, Santa Clara, CA, USA) on a DAKO Autostainer Link48 was carried out according to the manufacturer’s protocol. DAKO substrate working solution was used as a chromogenic agent for 2 × 5 min, and sections were counterstained with EnVision™ FLEX haematoxylin. Sections known to stain positively were included in each batch, and negative controls were prepared by replacing the primary antibody with the DAKO antibody diluent.

Individual biomarker expression was assessed by two independent observers (SW and MAB), one of whom was a trained gastrointestinal pathologist. Both observers were blinded to clinical data. Nuclear, cytoplasmic or cell membrane staining was considered, and ERα and ERβ receptor expression was recorded by calculating H-scores. The H-score incorporated staining intensity and frequency, with consensus agreement of discordant results. Scoring was based on intensity (0 = no staining, 1 = weak, 2 = moderate and 3 = strong staining observed) and percentage of tumour cells staining positive. These two values were multiplied to give an H-score between 0 and 300 for the section. A dichotomous classification was initially used to categorise H-scores into high (score 201–300), moderate (score 101–200), low (score 1–100) or negative expression. In the ERβ (ESR2) IHC population, the upper quartile H-score cut-off value was 100; therefore, for subsequent analysis, the moderate and high expression groups were combined. There was a good correlation between IHC H-score and Allred Score (Pearson R = 0.868 (95% CI; 0.828–0.899), *p* < 0.0001). An H-score of 100 equates to an Allred Score of 6.

Differences between clinicopathological characteristics according to ERα and ERβ expression were calculated using chi-squared tests with correction for multiple testing. Associations between sex hormone receptor expression groups and progression-free (PFS) and overall survival (OS) were investigated using Cox proportional hazards regression, producing unadjusted and adjusted hazard ratios (HR) and 95% confidence intervals (CIs). All analyses were adjusted for the GO2 study stratification factors; age group at randomisation, sex, stage, primary site, and dose level administered. All statistical analysis was performed using R statistical software (version 4.0.2; R Core Team 2021, Vienna, Austria. Available online: https://www.R-project.org/ accessed on 27 April 2023).

## 3. Results

### 3.1. Patient Cohort

**Figure 1:**
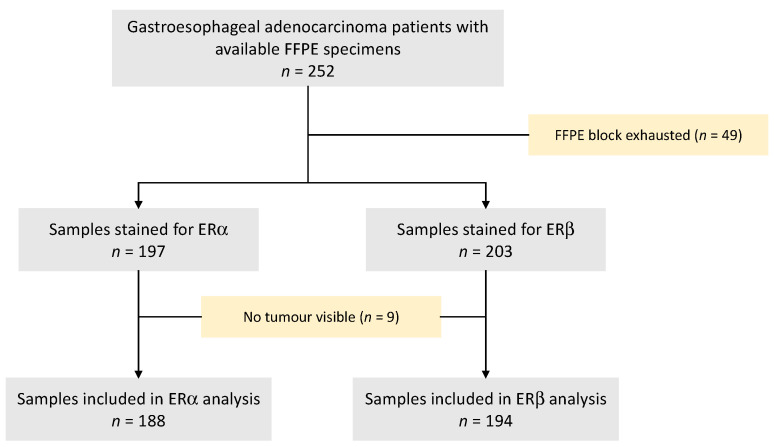
CONSORT diagram of GO2 patient selection for ERα and ERβ immunohistochemistry. ER—oestrogen receptor; FFPE—formalin-fixed paraffin-embedded.

FFPE blocks from 252 patients with advanced GOA were available. From these, 49 had already been exhausted, and therefore 203 FFPE blocks were suitable for immunohistochemical staining. ERβ was performed first, followed by ERα with tumours visible in 194 (95.6%) and 188 (92.6%) samples, respectively (Figure 1).

#### 3.1.1. ERα and ERβ Expression

For ERα, 187 (99.5%) of the 188 samples had no visible expression. One (0.5%) sample had low-intensity staining in 50% of visible tumour cells. Subsequent analysis relating to demographics and survival was not performed.

**Figure 2:**
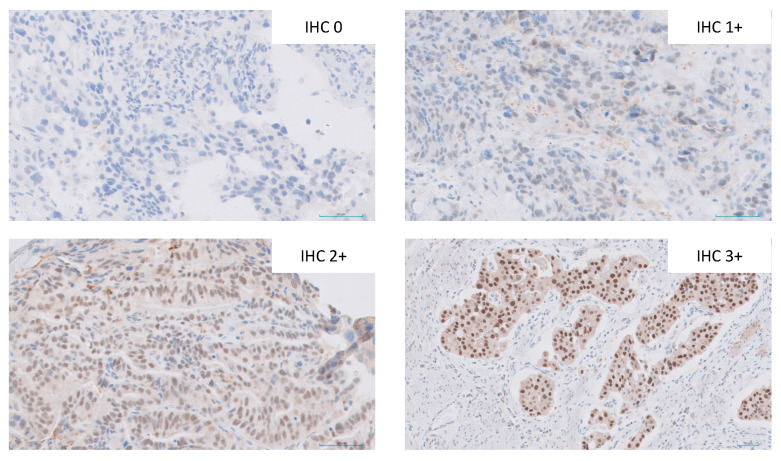
Examples of immunohistochemical staining intensity (×40 magnification) of ERβ from samples included in the study.

Examples of ERβ staining intensity are shown in Figure 2. Of the 194 samples available for ERβ analysis, the ERβ positivity rate was 70.6%; 57 (29.4%) had no expression, 98 (50.5%) had low expression (H-score 1–100), 35 (18.0%) had moderate expression (H-score 101–200) and 4 (2.1%) had high expression (H-score 201–300).

Demographics according to expression level are shown in Table 1. The median age of the population was 76 (range 52–90), and 25.3% were female. There were no differences in age, sex distribution, ECOG PS, site of primary or the presence/absence of metastasis according to expression level group. Despite the lack of significant difference in sex distribution between expression groups, female patients had lower rates of any ERβ expression than males, although this did not reach significance (61.2% vs 73.8%, *p* = 0.137). ERβ was expressed in significantly fewer patients aged younger than 65 (46.7%) than in the 65–75 cohort (78.3%) and the older than 75 cohort (69.1%) (*p* = 0.045) (Table 2). Expression was similar irrespective of the site of the primary tumour.

#### 3.1.2. ERβ Expression and Survival

**Figure 3:**
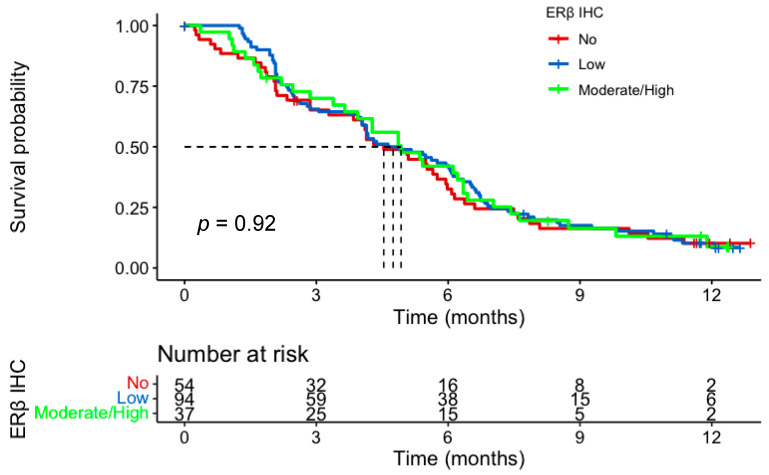
Progression-free survival according to ERβ IHC expression in the advanced gastroesophageal adenocarcinoma GO2 population who received at least one cycle of chemotherapy. Groups based on histoscore: no, 0; low, 0–100; moderate/high, 101–300.

**Figure 4:**
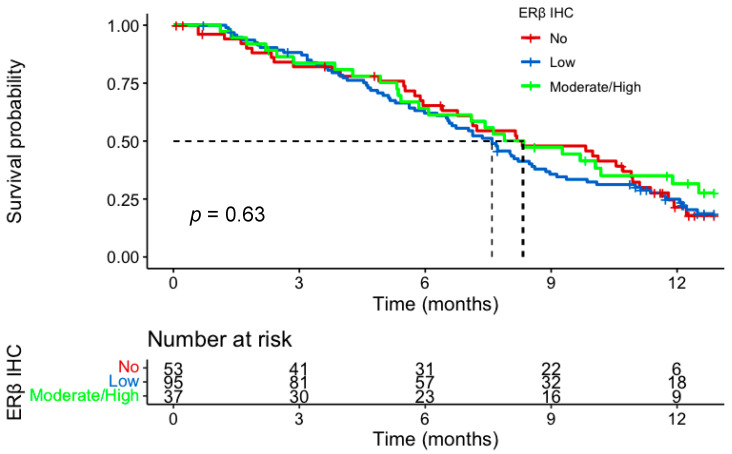
Overall Survival according to ERβ IHC expression in the advanced gastroesophageal adenocarcinoma GO2 population who received at least one cycle of chemotherapy. Groups based on histoscore: no—0; low, 0–100; moderate/high, 101–300.

**Figure 5:**
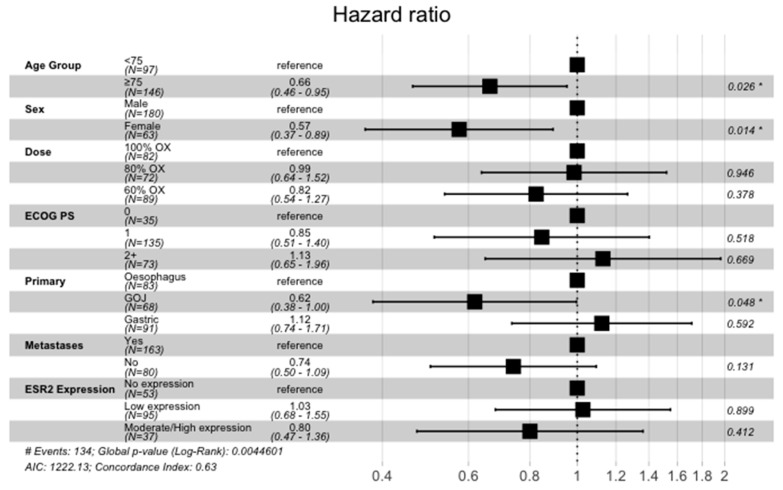
Cox regression analysis incorporating ERβ (labelled ESR2) IHC expression and stratification factors in the advanced gastroesophageal adenocarcinoma GO2 population who received at least one cycle of chemotherapy. * *p*-value < 0.05.

In the IHC cohort, 185 of the 194 patients received at least one cycle of chemotherapy and were included in the survival analysis. There was no evidence of a prognostic role for ERβ IHC expression for either PFS or OS. Median PFS for no, low, and moderate/high expression groups was 4.5 months (m) (95% CI; 3.8–6.0), 4.8 m (95% CI; 4.0–6.11) and 4.9 m (95% CI; 3.9–6.4), respectively. Median OS for the three groups was 8.3 m (95% CI; 6.8–10.9) vs 7.6 m (95% CI; 6.5–8.6) vs 8.3 m (95% CI; 6.1–12.5) (Figure 3 and Figure 4). There was no impact of chemotherapy dose level on survival in either test of interaction with IHC expression or on Cox-regression analysis (Figure 5). Of note, in the tested population, female sex (HR, 0.57; 95% CI 0.37–0.89; *p* = 0.014) and older age (HR, 0.66; 95% CI 0.46–0.95; *p* = 0.026) were good prognostic factors. This was also the case in the intention to treat GO2 adenocarcinoma population (HR, 0.73; *p* = 0.027).

## 4. Discussion

Gastroesophageal cancer is more common in males, and female sex is associated with improved outcomes. It has been proposed that this observation could be related to the effect of oestrogen on signalling via oestrogen receptors. This has been investigated previously, but mainly in younger populations treated with curative intent. In this study, we investigated tumour IHC expression of the oestrogen receptors α and β in a population of older adults with advanced GOA. We also investigated the prognostic role of this expression in patients treated with palliative chemotherapy. This is the largest report to date on this topic in an advanced setting and also provides valuable data on an older patient population.

We found that only one of the 188 (0.5%) patients expressed ERα. In contrast, 70.4% of samples had ERβ expression. There were no clear demographic associations with expression level; however, females had a lower proportion of tumours with ERβ expression. Survival analysis according to ERα expression was not possible, but there was no evident relationship between ERβ expression and either PFS or OS with palliative chemotherapy. Of note, female sex and older age were favourable prognostic markers in Cox regression analysis, and ERβ expression appeared to differ according to age.

Our ER IHC findings are in keeping with previous studies in both oesophageal and gastric adenocarcinoma (Table 3). Expression of ERα in published literature ranges from 0–40%, while ERβ expression ranges from 31–100% [20,21,22,23,24,25,26,27,28,29,30,31,32,33]. One of the main challenges in comparing studies is that a cut-off for ER IHC positivity is not yet defined in gastric cancer. There is also a range of scoring methods used, including the Allred scoring system used in breast cancer [34]. We attempted to address this challenge by using histoscore, which enabled any positivity, and strength of positivity to be explored separately.

For ERβ, we observed numerically lower expression in females in our cohort. This has been reported previously in other studies of gastric cancer. Ryu et al., in a study of 148 gastric cancers, reported a positivity rate of 61.2% in males compared to 38.8% in females, *p* = 0.931 [32]. In a larger study of 823 patients by Gan et al., the positivity rate was 92.7% in males vs 89.8% in females, *p* = 0.166. The higher rates of ERβ expression in males may suggest a potential biological role for oestrogen and/or ERβ in the development and progression of GOA.

In contrast, differences in expression according to sex have not been seen in oesophageal adenocarcinoma, but published studies are of smaller size. In the largest to date, McMenamin et al. explored expression in 138 tumours, the majority of which were gastroesophageal junctional (84.1%). Although the majority of positive expression in the cohort was seen in males, the rates of positivity were 31.5% in males compared to 30% in females [20].

The observation of lower ERβ expression in samples from younger patients has also been reported by Ryu et al.; however, an age cut-off of 50 years was used; 26.9% vs. 73.1%, *p* = 0.027 [32]. Gan et al. reported a positivity rate of 93.9% in patients aged 65 and older compared to 87.7% in those younger than 40. There was no difference in ERβ expression with age in McMenamin et al., with a positivity of 31.3% in those aged 70 and older compared to 28.6% in those younger than 50 [20].

When considering expression, it is important to consider that the findings presented in this study are in a population which is different in terms of age, fitness and stage of disease to previous literature. It is increasingly recognised that cancer disease biology differs with age [19], and it is possible, therefore, that expression of IHC biomarkers may differ in the GO2 population.

In our study, there was no impact of ERβ IHC expression on either PFS or OS within the trial population. This finding is in keeping with most previously published studies. The only exception is a study by Xu et al. that observed a survival benefit for ERβ expression in 211 patients with gastric cancer treated with curative intent [28]. Importantly the study population differed from ours; the mean age of the ERβ-positive and negative populations was 56.4 and 57.5 years, respectively, and the ERβ-negative cohort had higher nodal positivity (64.5% vs. 54.8%).

Our observation of improved survival with female sex in both the IHC population and the GO2 intention to treat population agrees with previously published data in both the curative and palliative settings [15,16,35]. The improved survival with older age, when controlled for other factors, supports the concept of differing disease biology with age in GOA.

The strengths of this study are that the samples were obtained from a clinical trial cohort. As such, the demographic and outcome data are reliable. In addition, this is a large sample size which is unique given the patient profile, stage of disease and standardised chemotherapy agents administered. An added strength is the ability to investigate the impact of chemotherapy dose level on outcome. The main limitation is that only one section of tumour was analysed for each receptor, and, as such, we were unable to mitigate the challenge of tumour heterogeneity.

In summary, this is the largest study of oestrogen receptor expression in an advanced gastroesophageal population. It also provides the first data on this topic, specifically in an older population. We demonstrate that ERα receptor expression is rare, while ERβ receptor expression occurs in most samples. IHC expression does not appear to impact survival; however, it may be influenced by patient age and/or sex. Female sex and older age were good prognostic factors in our population.

## 5. Conclusions

In this post hoc biomarker analysis of a completed clinical trial in older adults with advanced gastroesophageal cancer, IHC expression of oestrogen receptor-α was rare, while expression of oestrogen receptor-β was common. Oestrogen receptor expression was not prognostic; however, female sex and older age were associated with improved outcomes.

## Figures and Tables

**Table 1:** Baseline demographics according to ERβ immunohistochemical expression group. OX—oxaliplatin/capecitabine. * False discovery rate correction for multiple testing.

	ESR2 IHC Expression Group			
	No Expression	Low Expression	Moderate/High Expression	*p*-Value
	(*N =* 57)	(*N =* 98)	(*N =* 39)	(q-Value) *
**Age Group**				
<65	8 (14.0%)	4 (4.1%)	3 (7.7%)	0.151
65–75	15 (26.3%)	39 (39.8%)	15 (38.5%)	(0.483)
>75	34 (59.6%)	55 (56.1%)	21 (53.8%)	
**Sex**				
Male	38 (66.7%)	76 (77.6%)	31 (79.5%)	0.241
Female	19 (33.3%)	22 (22.4%)	8 (20.5%)	(0.483)
**ECOG PS**				
0	8 (14.0%)	10 (10.2%)	8 (20.5%)	0.434
1	31 (54.4%)	62 (63.3%)	19 (48.7%)	(0.652)
2+	18 (31.6%)	26 (26.5%)	12 (30.8%)	
**Dose Level**				
100% OX	20 (35.1%)	37 (37.8%)	9 (23.1%)	0.2
80% OX	16 (28.1%)	32 (32.7%)	10 (25.6%)	(0.483)
60% OX	21 (36.8%)	29 (29.6%)	20 (51.3%)	
**Primary Site**				
Oesophagus	19 (33.3%)	34 (34.7%)	10 (25.6%)	0.779
GOJ	18 (31.6%)	29 (29.6%)	11 (28.2%)	(0.827)
Gastric	20 (35.1%)	35 (35.7%)	18 (46.2%)	
**Metastasis present**				
Metastasis	36 (63.2%)	64 (65.3%)	27 (69.2%)	0.827
No metastasis	21 (36.8%)	34 (34.7%)	12 (30.8%)	(0.827)
**GO2 Frailty Group**				
No/mild frailty	7 (12.3%)	15 (15.3%)	11 (28.2%)	0.109
Moderate frailty	19 (33.3%)	22 (22.4%)	6 (15.4%)	(0.422)
Severe frailty	31 (54.4%)	61 (62.2%)	22 (56.4%)	

**Table 2:** ERβ IHC expression according to age group in the GO2 gastroesophageal adenocarcinoma population. * False discovery rate correction for multiple testing.

	Age Group			
	<65 (*N =* 15)	65–75 (*N =* 69)	>75 (*N =* 110)	*p*-Value (q-Value) *
**ERβ expression**				
No expression	8 (53.3%)	15 (21.7%)	34 (30.9%)	0.045
Positive expression	7 (46.7%)	54 (78.3%)	76 (69.1%)	−0.045

**Table 3:** Studies of ERα and ERβ IHC expression and outcome in gastroesophageal cancer. ER—oestrogen receptor, OAC—oesophageal adenocarcinoma.

Author	Year	Site	Age	Setting	Number	ERα	ERβ	Survival
Oesophageal Cancer								
McMenamin [20]	2018	EAC	Mean 63	Radical	139	4%	31%	ERα—no impact on survival ERβ—non-significant improvements
Kalayarasan [21]	2008	GOA	Mean 57.6	All stages	15	0%	100%	-
Al-Khyatt [22]	2018	OAC	Median 65 (range 30–79)	Radical	28	2.9%	41.2%	ERα and ERβ—poorer survival
Liu [23]	2004	OAC	Not available	Radical	27	-	ERβ1 85% ERβ2 81% ERβ3 100% ERβ5 100%	-
Akgun [24]	2002	OAC	Not available	Radical	23	-	100%	-
Tiffan [25]	2003	OAC	Range 29–90	Radical	20	40%	-	-
Gastric Cancer								
Tang [26]	2017	Gastric	Median 58	All stages	150/153	6%	93.5%	ERα—poorer survival ERβ—non-significant poorer survival
Gan [27]	2012	Gastric	Not available	All stages	848/823	12%	91.9%	ERα—improved survival ERβ—no impact on survival
Xu [28]	2010	Gastric	Mean 57 Range 31–79	Radical	211	25.6%	49.3%	ERα—poorer survival ERβ—better survival
Da Silva [29]	2022	Gastric	Mean 62.4	All stages	345	1.8%	98.2%	ERα—no impact on survival ERβ—no impact on survival
Wang [30]	2007	Gastric	Median 60 (range 32–87)	All stages	39	18.2%	43.6%	-
Zhou [31]	2016	Gastric	All under 40 (mean 33.8)	Radical	139	49.6%	87.8%	ERα—no impact on survival ERβ—no impact on survival
Ryu [32]	2012	Gastric	Unknown	Radical	148	-	45.3%	ERβ—no impact on survival
Jukic [33]	2017	Gastric	Mean 69 Range 35–90	Radical	60	20%	-	-

## Data Availability

Data and script can be obtained on request from the corresponding author.
